# Supplementary figures and images for: Research on optimization of transportation routes for infectious medical waste
Source: PLoS One. 2025 Sep 26;20(9):e0330996. doi: 10.1371/journal.pone.0330996 (PMC12469087; doi:10.1371/journal.pone.0330996)

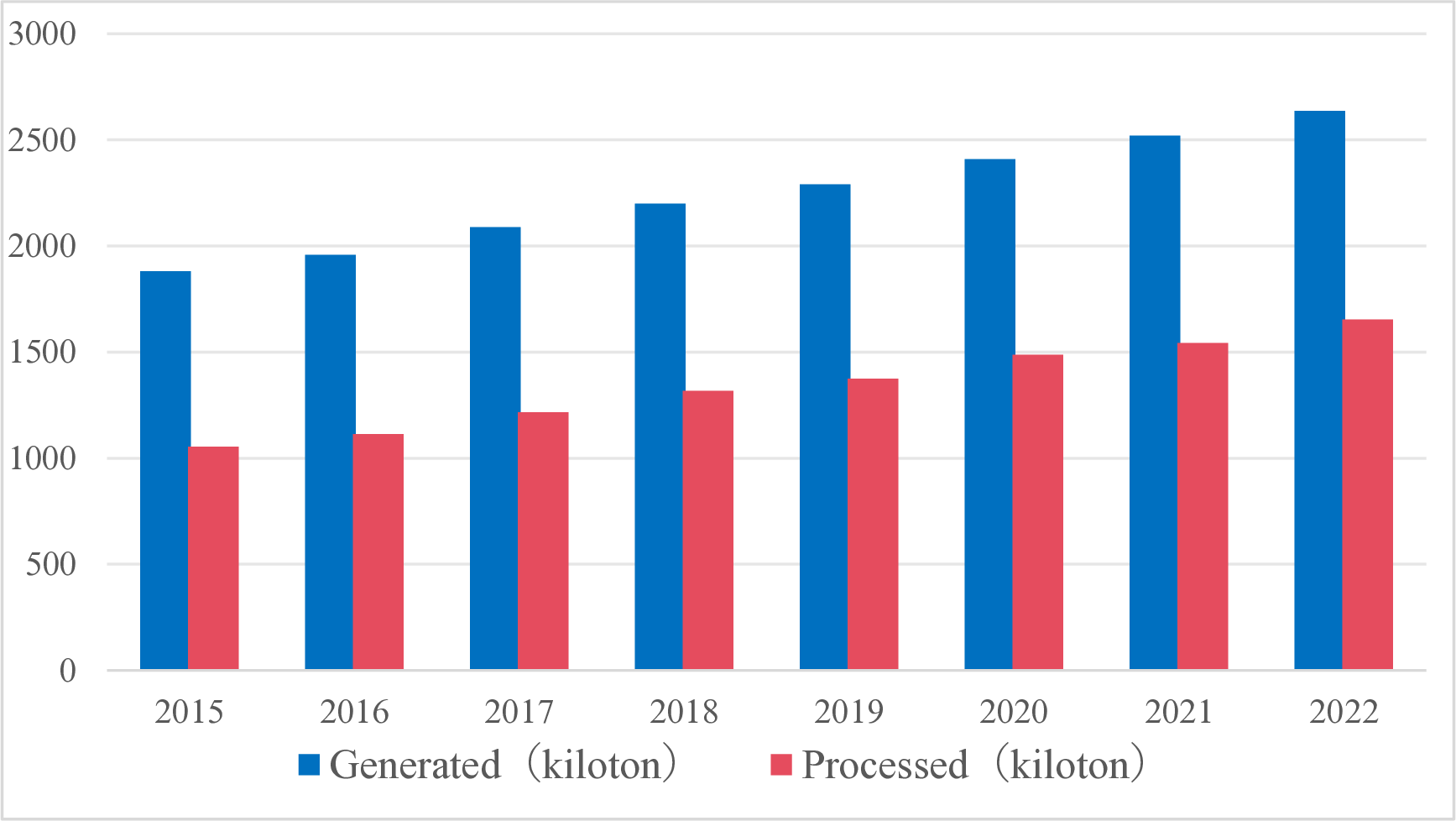

Supplement: S1 Fig — (TIF) [file pone.0330996.s001.tif]

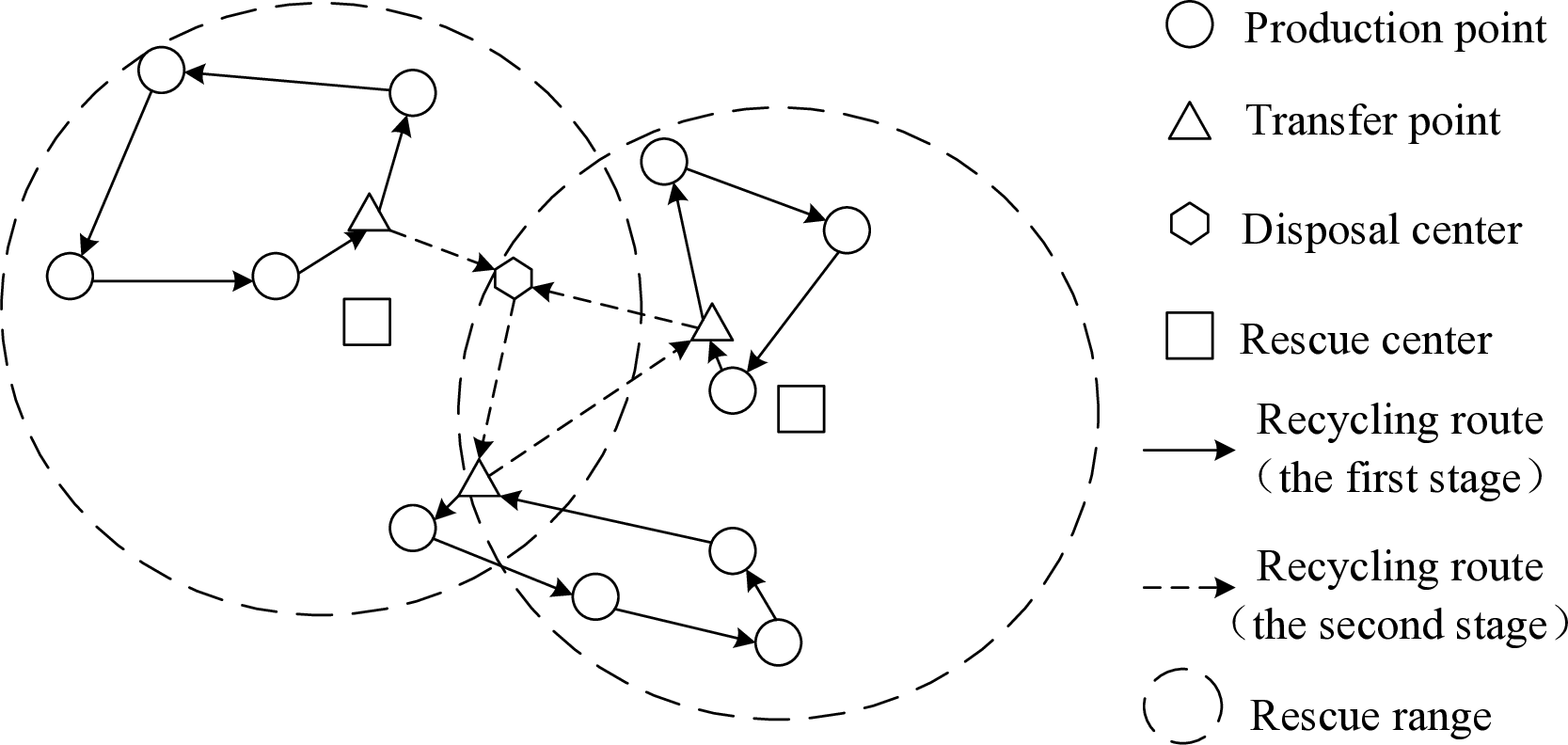

Supplement: S2 Fig — (TIF) [file pone.0330996.s002.tif]

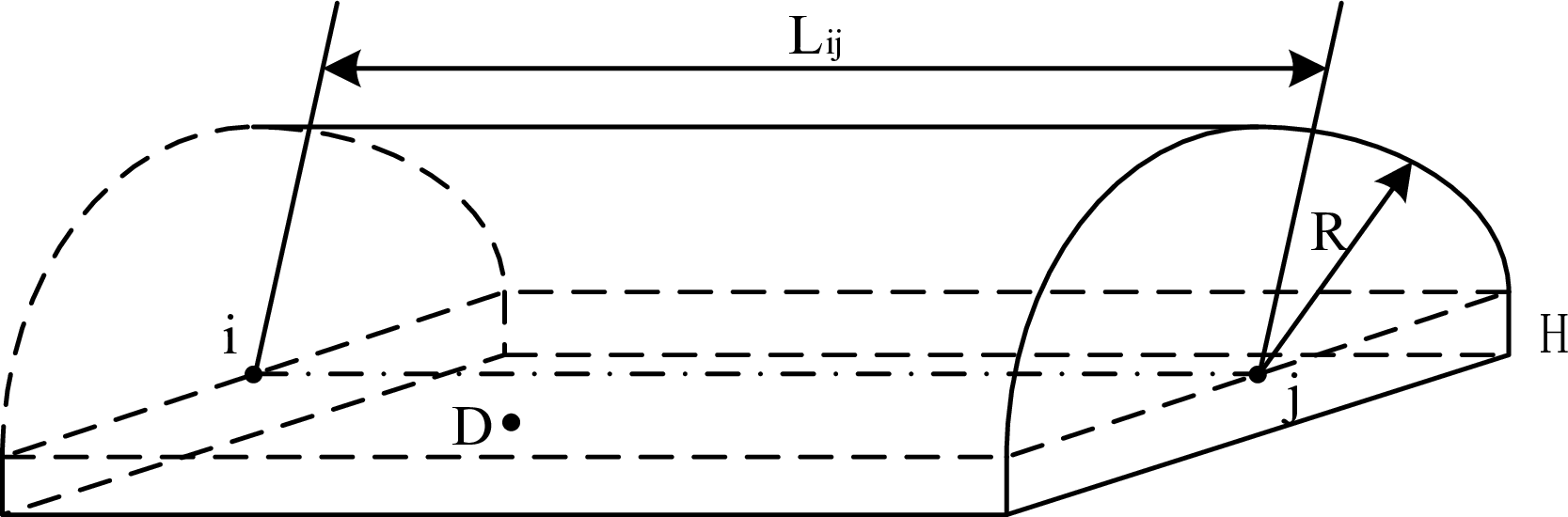

Supplement: S3 Fig — (TIF) [file pone.0330996.s003.tif]

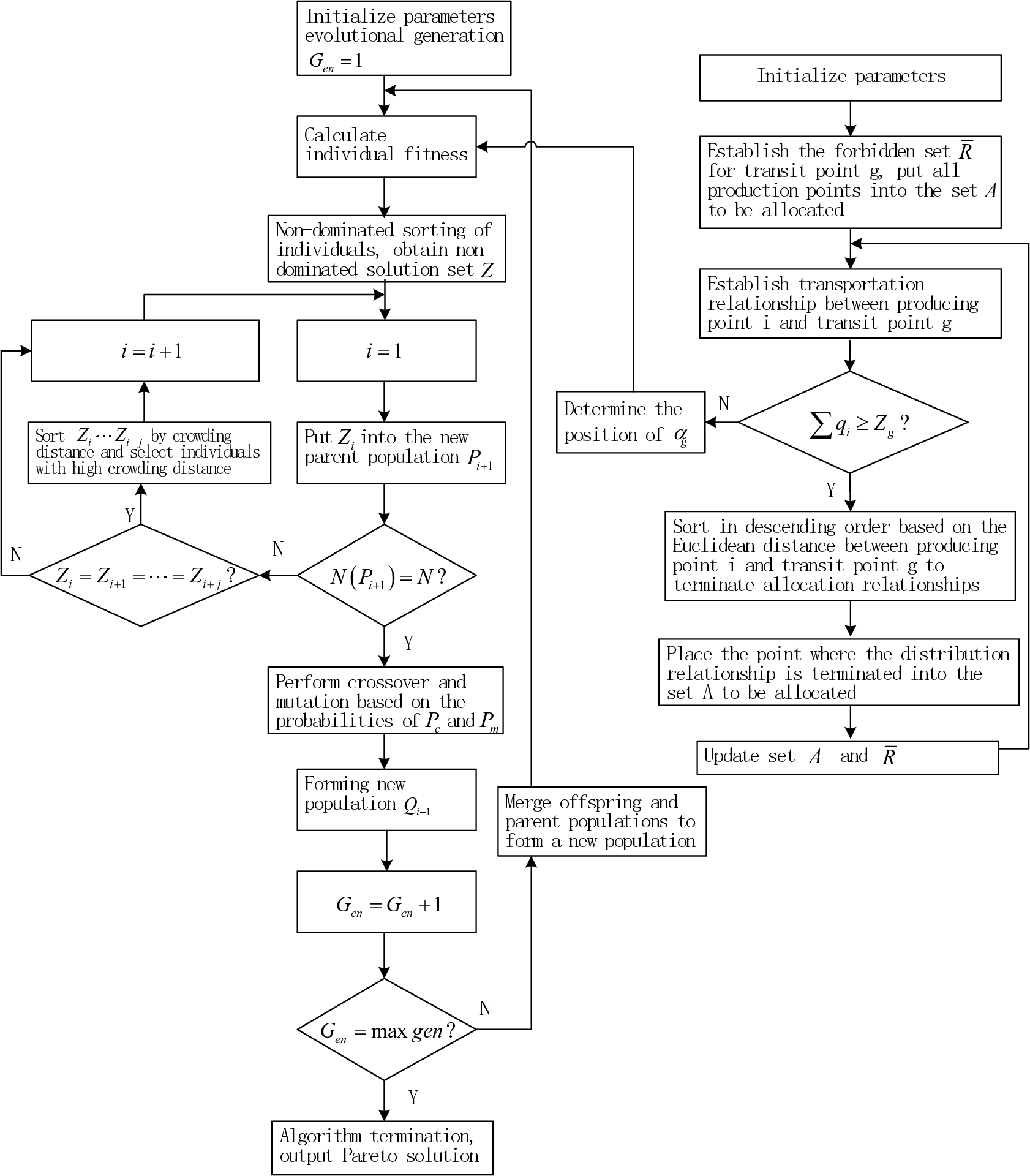

Supplement: S4 Fig — (TIF) [file pone.0330996.s004.tif]

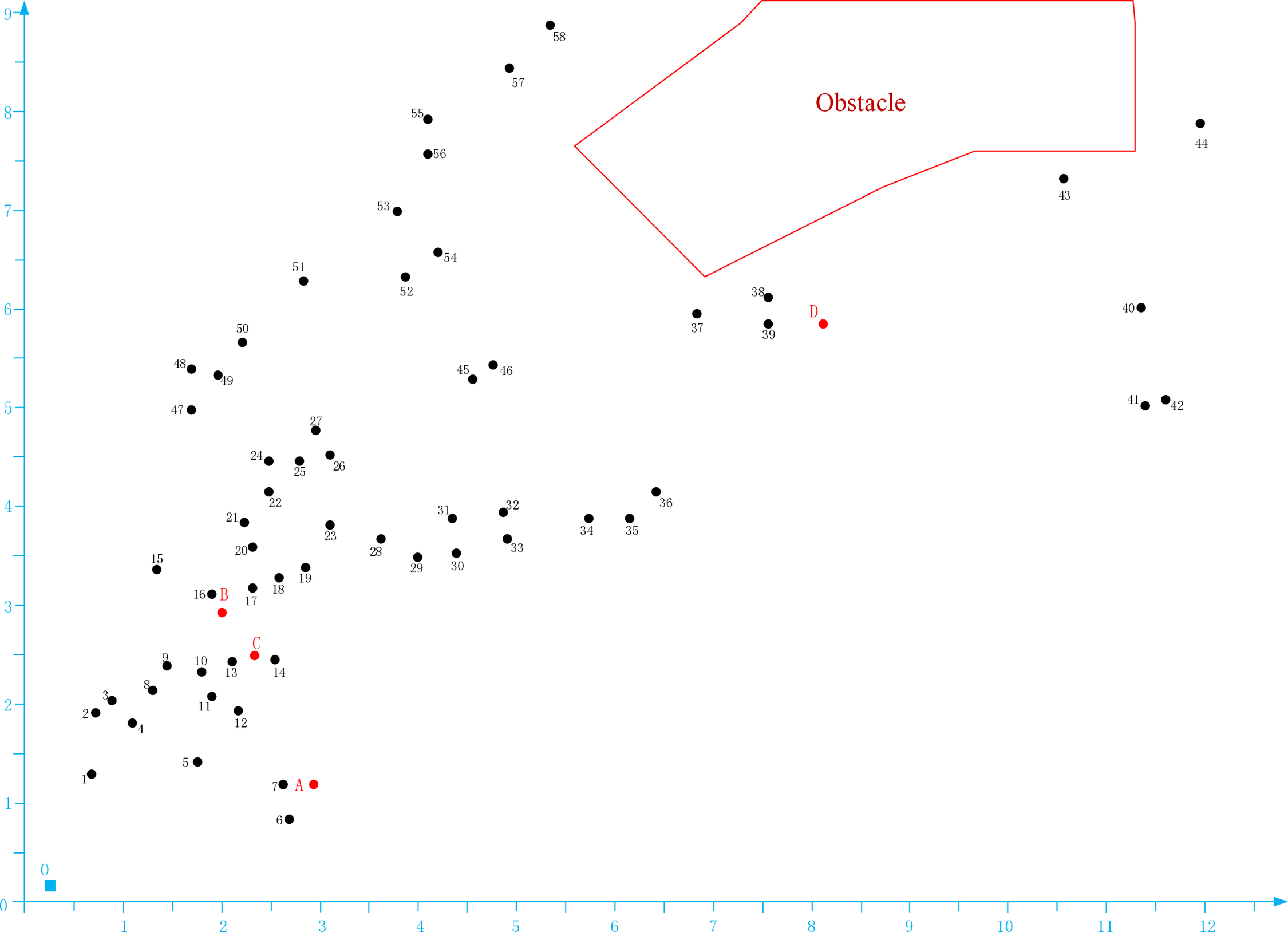

Supplement: S5 Fig — (TIF) [file pone.0330996.s005.tif]

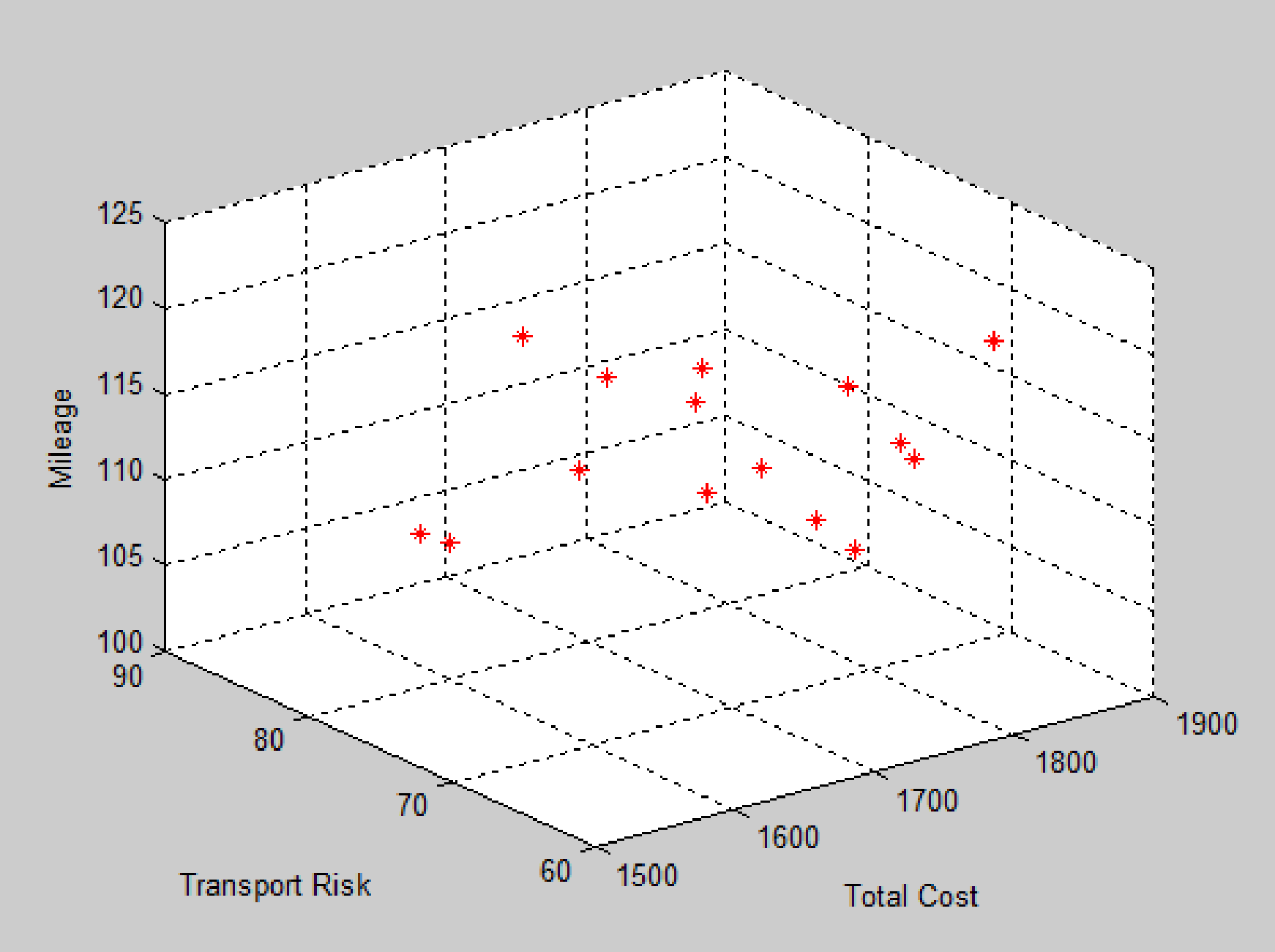

Supplement: S6 Fig — (TIF) [file pone.0330996.s006.tif]

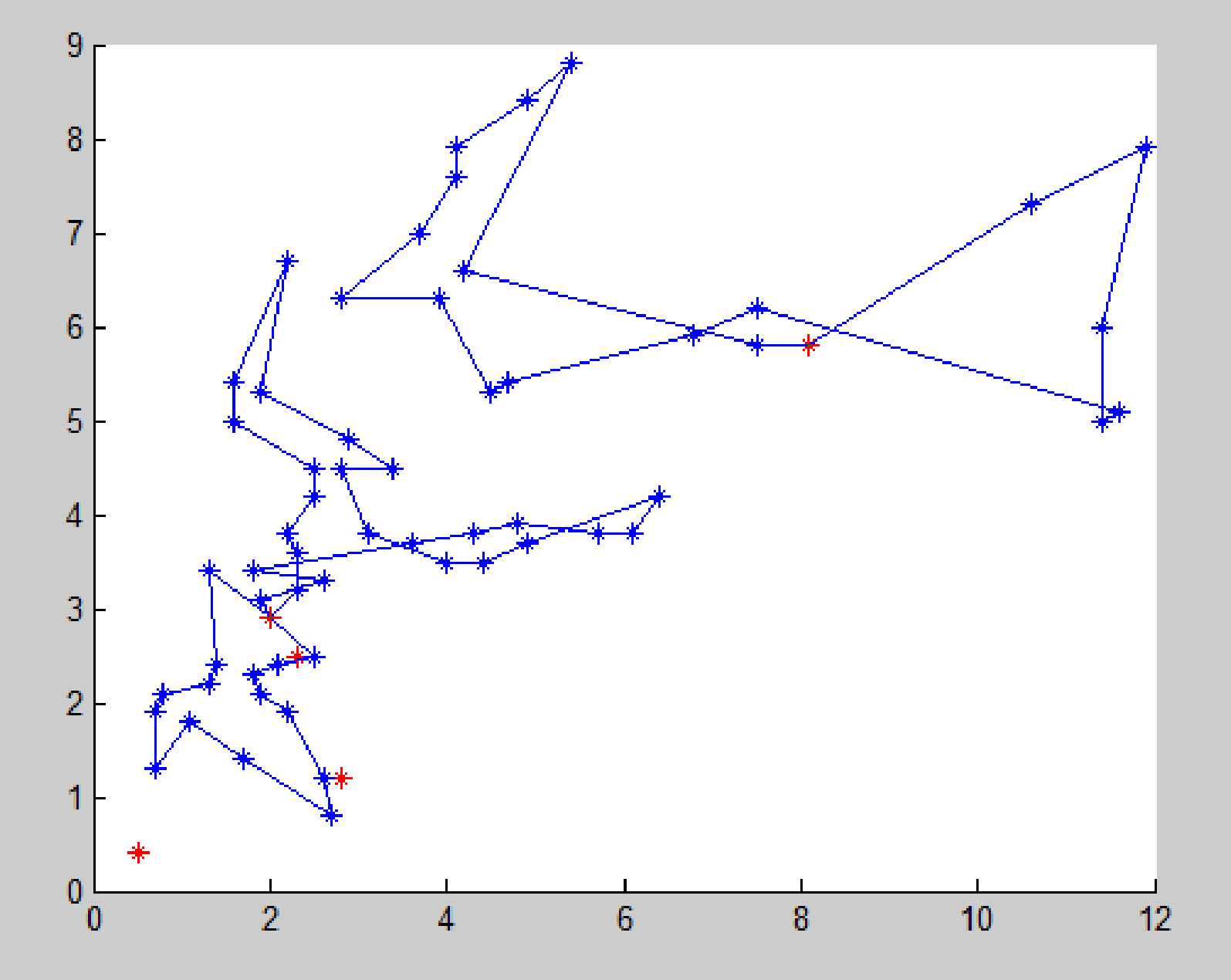

Supplement: S7 Fig — (TIF) [file pone.0330996.s007.tif]

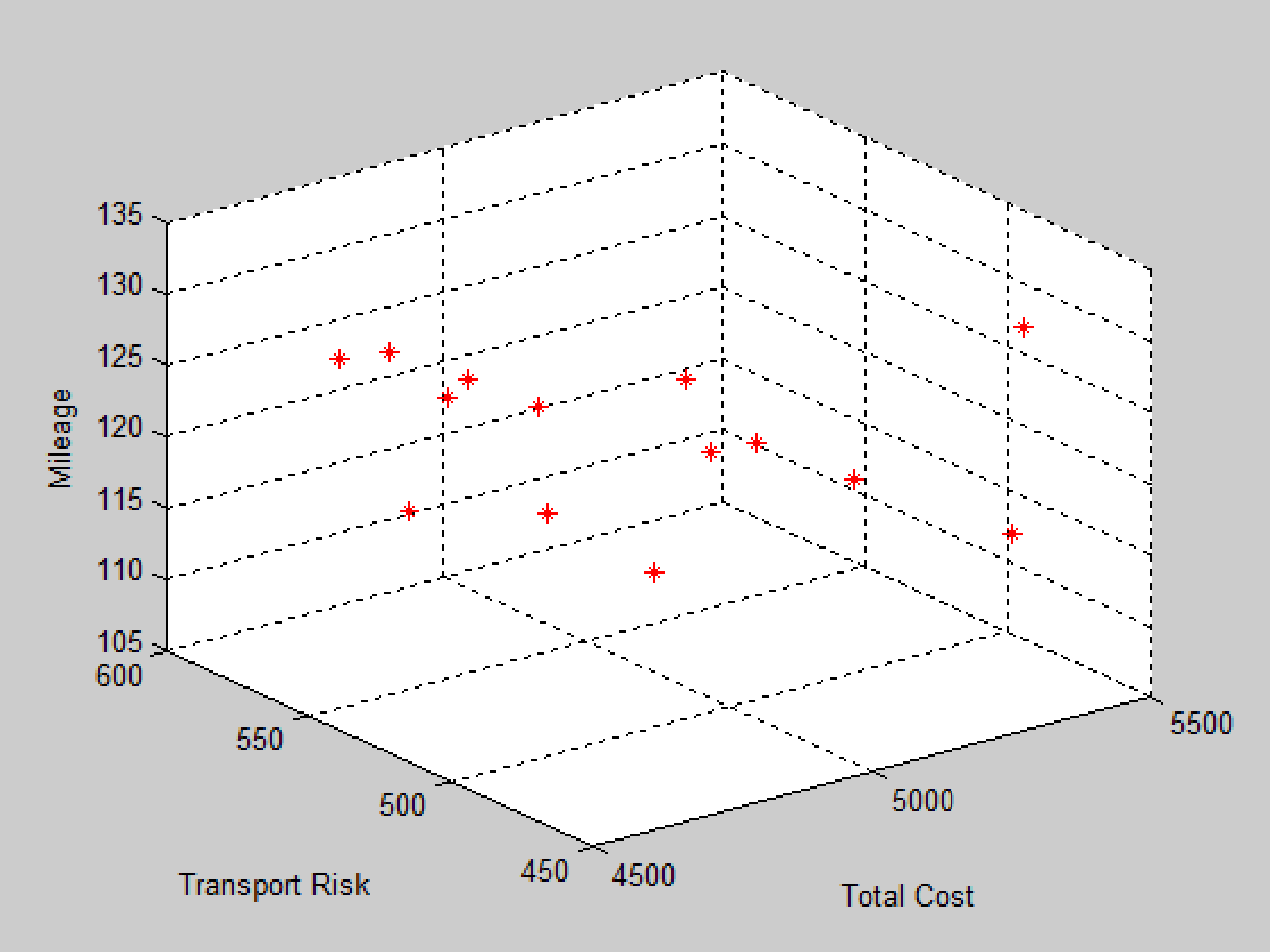

Supplement: S8 Fig — (TIF) [file pone.0330996.s008.tif]

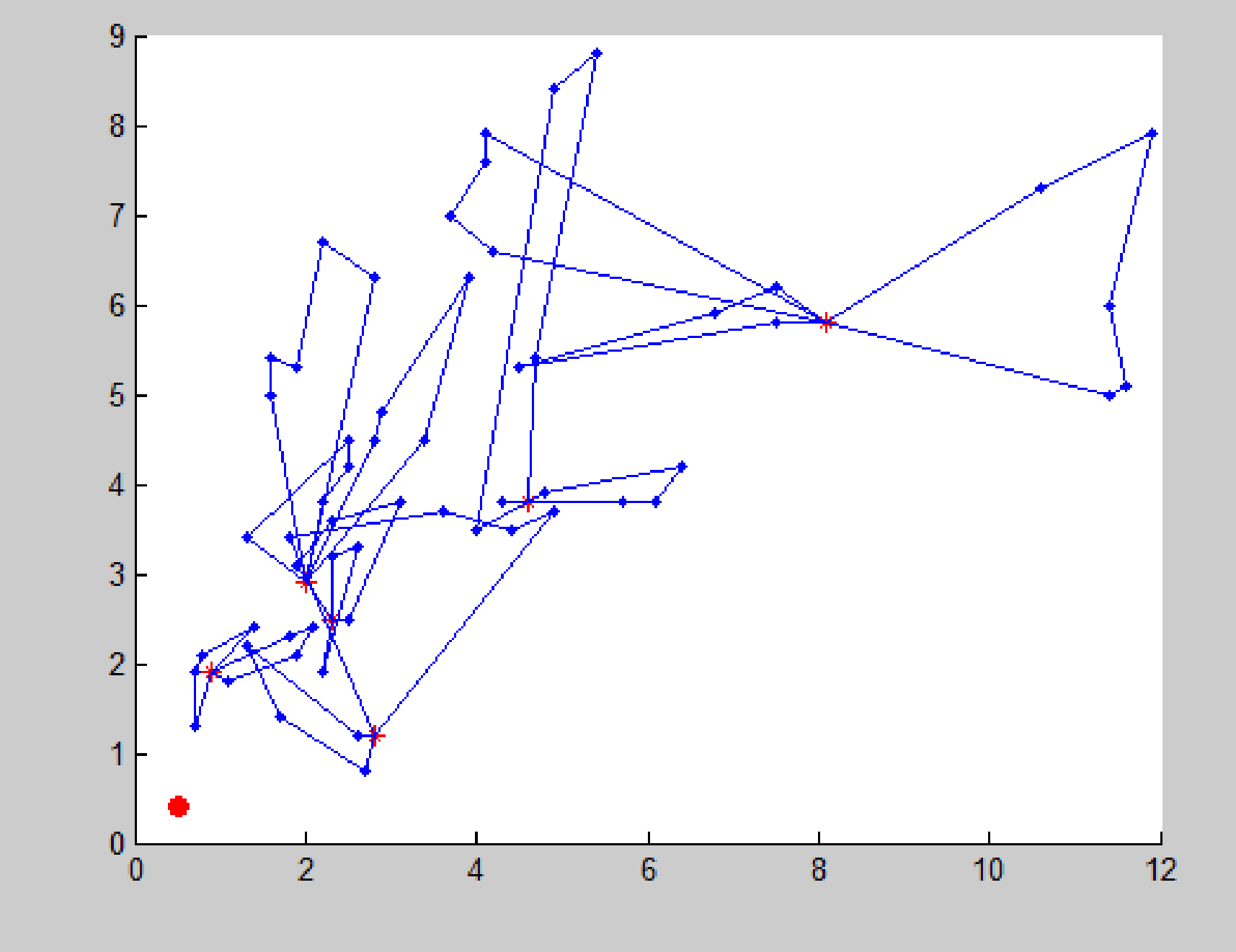

Supplement: S9 Fig — (TIF) [file pone.0330996.s009.tif]

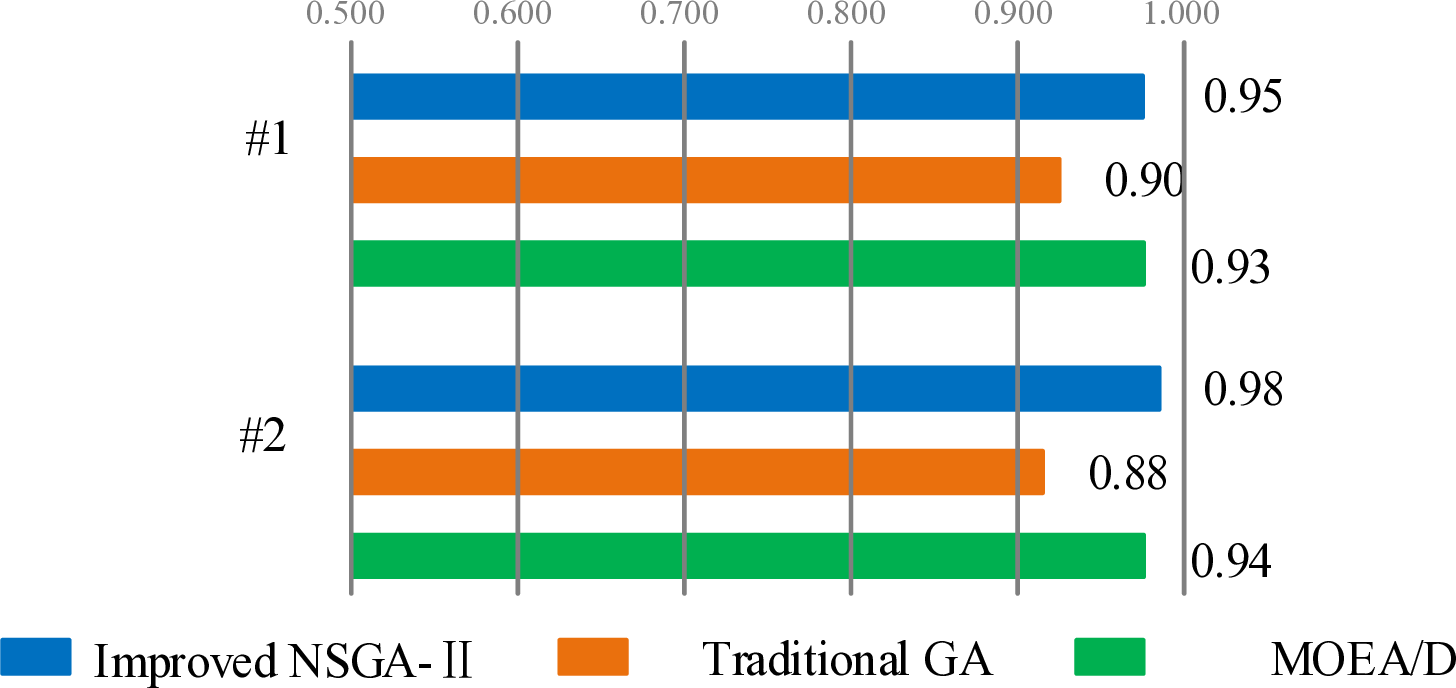

Supplement: S10 Fig — (TIF) [file pone.0330996.s010.tif]
