# Supplementary material for: Research on optimization of transportation routes for infectious medical waste
Source: PLoS One. 2025 Sep 26;20(9):e0330996. doi: 10.1371/journal.pone.0330996 (PMC12469087; doi:10.1371/journal.pone.0330996)
Supplement: S1 Table — (DOCX) [file pone.0330996.s011.docx]

**Tab.1** **Comparison ofresearch contributions withexisting studies**

| **Authors** | **Cost** | **Capacity** | **Risk** | **Mileage** | **Objective** | **Method** | **Environment** |
| --- | --- | --- | --- | --- | --- | --- | --- |
| **Stancu (1976)** | **√** | √ |  |  | Multi | MILP | Deterministic‌ |
| **Stern (1981)** |  |  |  | √ | Single | MILP | Deterministic‌ |
| **Listers (2005)** | √ |  |  |  | Single | MILP | Stochastic |
| **Aylin (2008)** | √ |  |  |  | Single | MILP | Stochastic |
| **Nie (2018)** | √ | √ |  |  | Multi | GA | Deterministic‌ |
| **Dong (2022)** | √ |  | √ |  | Multi | GA | Deterministic‌ |
| **Shi (2011)** |  |  |  | √ | Single | ACS | Deterministic‌ |
| **He (2007)** | √ |  | √ |  | Multi | Greedy | Deterministic‌ |
| **Osaba (2018)** | √ |  |  | √ | Multi | Bat | Deterministic‌ |
| **Le (2016)** | √ |  |  | √ | Multi | MILP | Deterministic‌ |
| **Liu (2016)** |  |  |  | √ | Single | Neighbor | Deterministic‌ |
| **Ma (2016)** | √ |  |  | √ | Multi | MILP | Deterministic‌ |
| **Alsh (2017)** | √ |  |  | √ | Multi | MILP | Stochastic |
| **Wang (2023)** | √ |  | √ |  | Multi | GA | Deterministic‌ |
| **This article** | √ | √ | √ | √ | Multi | NSGA-II | Deterministic‌ |
