# Supplementary material for: Research on optimization of transportation routes for infectious medical waste
Source: PLoS One. 2025 Sep 26;20(9):e0330996. doi: 10.1371/journal.pone.0330996 (PMC12469087; doi:10.1371/journal.pone.0330996)
Supplement: S2 Table — (DOCX) [file pone.0330996.s012.docx]

**Tab. 2Symbols used in the paper**

| Symbols | Parameter |
| --- | --- |
| $N=V\cup G\cup S$ | Node set |
| $V=\{1,\cdots,i,\cdots\text{,}j,\cdots\}$ | Node set of producing points |
| $G=\{1,\cdots,g,\cdots,g',\cdots\}$ | Node setofbackup transfer points |
| $S=\{1,\cdots,s,\cdots,s',\cdots\}$ | Node setof disposal center |
| $K=\{1,\cdots,k,\cdots\}$ | Node setof vehicles |
| E | Set of point-to-point route |
| $D=\{1,\cdots,d\}$ | Location of rescue center(handling infectious incidents) |
| H | Vehicle height |
| R | Infection radius |
| $\mu$ | Diffusion velocity |
| T | Diffusion time |
| $d_{Dij}$ | The shortest distance from rescue center route$(i,j)$ |
| $v_{D}$ | Average speed of rescue vehicles |
| $Q_{ij}$ | The weight of medical waste infected on route $(i,j)$ |
| $F_{D}$ | The rescue capabilities that rescue center D can provide |
| $L_{ij}$ | Length of route $(i,j)$ |
| $O_{ij}$ | Spread range of the virus on route $(i,j)$ |
| $\theta$ | Probability of medical waste carrying infectious viruses |
| $P_{ij}$ | Population density on the route$(i,j)$ |
| M | Population within the scope of virus spread |
| $TR_{ij}$ | Transportation risks on the route$(i,j)$ |
| $FC_{g}$ | Construction cost of the transfer center g |
| $FC_{k}$ | Fixed transportation cost of vehicle k |
| $q_{i}$ | The weight of medical infections produced in medical institution i |
| $q_{g}$ | The weight of medical infectious materials stored in transfer center g |
| $Z_{k}$ | Maximum capacity of vehicle k |
| $Z_{g}$ | Maximum capacity of transfer center g |
| $Z_{s}$ | Maximum capacity of the disposal center s |
| $C$ | Unit transportation cost |
| $R_{D}$ | Rescue radius of rescue center D |
| $\alpha_{g}$ | 0-1 variable, If a transfer point is established at node g,$\alpha_{g}$=1, otherwise, $\alpha_{g}$=0 |
| $\beta_{ij}$ | 0-1 variable, If the vehicle passes through route$(i,j)$,$\alpha_{g}$=1, otherwise, $\alpha_{g}$=0 |
| $\gamma_{k}$ | 0-1 variable, If vehicle k is used for transportation,$\alpha_{g}$=1, otherwise, $\alpha_{g}$=0 |
