# Supplementary material for: Research on optimization of transportation routes for infectious medical waste
Source: PLoS One. 2025 Sep 26;20(9):e0330996. doi: 10.1371/journal.pone.0330996 (PMC12469087; doi:10.1371/journal.pone.0330996)
Supplement: S3 Table — (DOCX) [file pone.0330996.s013.docx]

**Tab.3** **Node coordinates and production**

| Node | X-axis | Y-axis | Production(t) | Node | X-axis | Y-axis | Production(t) | Node | X-axis | Y-axis | Production(t) |
| --- | --- | --- | --- | --- | --- | --- | --- | --- | --- | --- | --- |
| O | 0.5 | 0.4 | $\infty$/$\infty$ | 17 | 2.3 | 3.2 | 38/255 | 38 | 7.5 | 6.2 | 36/181 |
| A | 2.8 | 1.2 | 2500 | 18 | 2.6 | 3.3 | 34/257 | 39 | 7.5 | 5.8 | 47/253 |
| B | 2.0 | 2.9 | 3200 | 19 | 1.8 | 3.4 | 26/296 | 40 | 11.4 | 6.0 | 33/144 |
| C | 2.3 | 2.5 | 2000 | 20 | 2.3 | 3.6 | 31/142 | 41 | 11.4 | 5.0 | 43/150 |
| D | 8.1 | 5.8 | 3000 | 21 | 2.2 | 3.8 | 33/305 | 42 | 11.6 | 5.1 | 33/246 |
| 1 | 0.7 | 1.3 | 31/259 | 22 | 2.5 | 4.2 | 46/154 | 43 | 10.6 | 7.3 | 27/194 |
| 2 | 0.7 | 1.9 | 40/232 | 23 | 3.1 | 3.8 | 40/262 | 44 | 11.9 | 7.9 | 38/259 |
| 3 | 0.8 | 2.1 | 42/293 | 24 | 2.5 | 4.5 | 46/313 | 45 | 4.5 | 5.3 | 50/232 |
| 4 | 1.1 | 1.8 | 43/252 | 25 | 2.8 | 4.5 | 38/159 | 46 | 4.7 | 5.4 | 46/257 |
| 5 | 1.7 | 1.4 | 31/267 | 26 | 3.4 | 4.5 | 38/321 | 47 | 1.6 | 5.0 | 49/216 |
| 6 | 2.7 | 0.8 | 47/297 | 27 | 2.9 | 4.8 | 43/202 | 48 | 1.6 | 5.4 | 44/154 |
| 7 | 2.6 | 1.2 | 27/154 | 28 | 3.6 | 3.7 | 41/179 | 49 | 1.9 | 5.3 | 39/174 |
| 8 | 1.3 | 2.2 | 45/278 | 29 | 4.0 | 3.5 | 48/150 | 50 | 2.2 | 5.7 | 27/309 |
| 9 | 1.4 | 2.4 | 37/215 | 30 | 4.4 | 3.5 | 34/277 | 51 | 2.8 | 6.3 | 37/141 |
| 10 | 1.8 | 2.3 | 29/265 | 31 | 4.3 | 3.8 | 36/310 | 52 | 3.9 | 6.3 | 44/305 |
| 11 | 1.9 | 2.1 | 37/191 | 32 | 4.8 | 3.9 | 50/188 | 53 | 3.7 | 7.0 | 37/307 |
| 12 | 2.2 | 1.9 | 31/159 | 33 | 4.9 | 3.7 | 45/237 | 54 | 4.2 | 6.6 | 40/291 |
| 13 | 2.1 | 2.4 | 34/193 | 34 | 5.7 | 3.8 | 29/132 | 55 | 4.1 | 7.9 | 29/248 |
| 14 | 2.5 | 2.5 | 27/276 | 35 | 6.1 | 3.8 | 45/187 | 56 | 4.1 | 7.6 | 39/249 |
| 15 | 1.3 | 3.4 | 43/225 | 36 | 6.4 | 4.2 | 35/147 | 57 | 4.9 | 8.4 | 28/264 |
| 16 | 1.9 | 3.1 | 43/302 | 37 | 6.8 | 5.9 | 46/322 | 58 | 5.4 | 8.8 | 34/314 |
