# Supplementary material for: Research on optimization of transportation routes for infectious medical waste
Source: PLoS One. 2025 Sep 26;20(9):e0330996. doi: 10.1371/journal.pone.0330996 (PMC12469087; doi:10.1371/journal.pone.0330996)
Supplement: S4 Table — (DOCX) [file pone.0330996.s014.docx]

**Tab.4** **Explanation and Values of Relevant Parameters in the Case**

| **Symbols** | **Parameter** | **Values** |
| --- | --- | --- |
| $\mu$ | Average wind speed | 4.43km/h |
| $\theta$ | Probability of waste being infectious | 0.6 |
| $P_{ij}$ | The population density of all lines in the region | 7500per/km^2^ |
| $H$ | Vehicle height | 1 meter |
| $FC_{g}$ | Cost of adding rescue centers | 50 million yuan |
| $F_{D}$ | The rescue capacity of the rescue center | 1000kg/h |
| $R_{D}$ | The coverage radius of the rescue center | 500 meters |
| $v_{D}$ | Driving speed | 40 km/h |
| $Z_{k,1}$ | Vehicle load capacity in first echelon | 5 t |
| $Z_{k,2}$ | Vehicle load capacity in second echelon | 1 t |
| $FC_{k,1}$ | Fixed cost in first echelon | 5million yuan |
| $FC_{k,2}$ | Fixed cost in second echelon | 2million yuan |
| $fc_{k,1}$ | Unit mileage transportation costin first echelon | 100 thousand yuan |
| $fc_{k,2}$ | Unit mileage transportation costin second echelon | 50 thousand yuan |
