# Supplementary material for: Research on optimization of transportation routes for infectious medical waste
Source: PLoS One. 2025 Sep 26;20(9):e0330996. doi: 10.1371/journal.pone.0330996 (PMC12469087; doi:10.1371/journal.pone.0330996)
Supplement: S5 Table — (DOCX) [file pone.0330996.s015.docx]

**Tab.5** **Delivery plan results for low medical waste production**

| **Optimal route** | **Path** | **Total**  **Cost** | **Transport**  **risk** | **Vehicle**  **mileage** | **Vehicle**  **Number** |
| --- | --- | --- | --- | --- | --- |
| **First Stage** | Path1_1  Path1_2  Path1_3 | 1063 | 73.12 | 92.62 | 3 |
| **Second Stage** | Path2_1 | 690 | 0 | 18.99 | 1 |
| **Total** |  | 1753 | 73.12 | 111.61 | 4 |
