# Supplementary material for: Research on optimization of transportation routes for infectious medical waste
Source: PLoS One. 2025 Sep 26;20(9):e0330996. doi: 10.1371/journal.pone.0330996 (PMC12469087; doi:10.1371/journal.pone.0330996)
Supplement: S6 Table — (DOCX) [file pone.0330996.s016.docx]

**Tab.6** **Delivery plan results for increased medical waste production**

| **Optimal route** | **Path** | **Total**  **Cost** | **Transport**  **risk** | **Vehicle**  **mileage** | **Vehicle**  **Number** |
| --- | --- | --- | --- | --- | --- |
| **First Stage** | A-7-8-5-6-A  A-19-28-30-33-A  B-15-24-22-21-B  B-47-48-49-50-51-B  B-25-27-52-26-B  C-16-20-23-14-C  C-17-18-12-C  D-55-56-53-54-D  D-43-44-40-42-41-D  D-38-37-45-39-D  E-9-3-2-1-E  E-10-13-11-4-E  F-32-36-35-34-31-F  F-46-58-57-29-F | 3249 | 517.26 | 89.78 | 14 |
| **Second Stage** | O-F-D-O  O-C-B-O  O-A-E-O | 1809 | 0 | 30.87 | 3 |
| **Total** |  | 5058 | 517.26 | 120.65 | 17 |
