# Supplementary material for: Research on optimization of transportation routes for infectious medical waste
Source: PLoS One. 2025 Sep 26;20(9):e0330996. doi: 10.1371/journal.pone.0330996 (PMC12469087; doi:10.1371/journal.pone.0330996)
Supplement: S7 Table — (DOCX) [file pone.0330996.s017.docx]

**Table 7.** **Comparison of calculation results**

|  | Improved NSGA-II | | MOEA/D | | Traditional genetic algorithm | | Average optimization rate |
| --- | --- | --- | --- | --- | --- | --- | --- |
| Objective function | low waste production | increased waste production | low waste production | increased waste production | low waste production | increased waste production |  |
| The amount of medical waste produced(kg) | 2209 | 13435 | 2209 | 13435 | 2209 | 13435 | / |
| Vehicle driving distance(km) | 111.61 | 120.65 | 111.61 | 130.24 | 120.23 | 134.58 | 8.76% |
| Number of vehicles | 4 | 17 | 4 | 18 | 5 | 19 | 12.78% |
| Total transportation cost(yuan) | 1753 | 5058 | 1753 | 5303 | 2037 | 5589 | 11.72% |
| Transportation risk(t·person/km^3^) | - | 517.26 | - | 520.58 | - | 527.97 | 2.03% |
| The full load rate of the vehicle | 55.23% | 92.66% | 55.23% | 90.12% | 49.09% | 86.68% | 6.06% |
