# Supplementary material for: Research on optimization of transportation routes for infectious medical waste
Source: PLoS One. 2025 Sep 26;20(9):e0330996. doi: 10.1371/journal.pone.0330996 (PMC12469087; doi:10.1371/journal.pone.0330996)
Supplement: Attachment — Submitted filename: 2.Response(3).docx [file pone.0330996.s018.docx]

**Original Manuscript ID:** **PONE-D-24-35149**

**Original Article Title: “Research on Optimization of Transportation Routes for Infectious Medical Waste”**

**To:** Plos One Editor

**Re:** Response to reviewers

Dear Editor,

Thank you for allowing a resubmission of our manuscript, with an opportunity to address the reviewers’ comments.

We are uploading (a) our point-by-point response to the comments (below) (response to reviewers), (b) an updated manuscript with yellow highlighting indicating changes, and (c) a clean updated manuscript without highlights (Word main document).

Best regards,

Hao Chen, Libo Li, Xuerui Qin*, Wenxian Wang.

**Reviewer 1 Comments to the Author**

My comments are as follows:

1. Can you elaborate on why a two-echelon model was chosen over other potential models? What specific advantages does it offer in the context of medical waste transportation?

According to the reviewer’s requirement, relevant statements have been added to in Section 3.1.

" From Figure 1, it can be seen that the infectious medical waste transportation system is architecture in above the urban road network containing emergency system， including the produce points (medical institutions), transfer points, and disposal centers of medical waste. In the first stage, transportation vehicles depart from the transfer point, collect medical waste from several production points, and then return to the transfer point for temporary storage; The second stage is similar to the first stage, starting from the disposal center, passing through several transfer centers, and returning to the disposal center. If a leakage accident occurs during transportation, the rescue center in the area will quickly handle it to control the spread of the virus. Therefore, the problem of transfer point location - transportation route decision is a two-stage multi-objective optimization problem, that involves collaborative optimization of multiple links such as rescue centers, medical institutions, transfer points, and disposal centers in the event of a virus leak."

2. The paper mentions a three-dimensional risk measurement sub-model. Could you provide more details on how this model was constructed and the specific parameters it includes?

According to the reviewer’s requirement, relevant statements have been added to in Section 3.2.

“It can be defined that vehicles transporting infectious medical waste are risk points, and the trajectory of the risk point is a line segment. Considering the characteristics of infectious viruses, their diffusion process can be described as follows: starting from vehicles, the virus spreads in a three-dimensional manner with a certain diffusion radius. Within a certain period of time, the virus will roughly form a region similar to a half cylinder, as shown in Figure 2. It can be found that the risk of virus transmission mainly depends on parameters such as spread radius $R$, transportation path length $L_{ij}$, and vehicle height $H$.”

According to the reviewer’s requirement, relevant statements have been added to in Section 4.2.2.

“The total transportation risk is represented by the product of transportation risk, diffusion time, and population density along each section of the route.

The radius of the diffusion range is:

$R\text{=}\mu\cdot T$（5）

Among them, the diffusion time $T$can be expressed as:

$T\text{=}\frac{d_{Dij}}{v_{D}}+\frac{Q_{ij}}{F_{D}}$（6）

The cross-sectional area of the diffusion range is:

$S\text{=}\frac{1}{2}\pi R^{2}+2R\cdot H$（7）

$L_{ij}$ is the length of the route$\left(i,j\right)$, and the volume of diffusion range can be expressed as:

$O_{ij}\text{=}S\cdot L_{ij}=\left(\frac{1}{2}\pi R^{2}+2R\cdot H\right)\cdot L_{ij}$（8）

The concentration of the virus is represented by the population within the current diffusion range:

$M=\pi\cdot\mu^{2}\cdot T^{2}\cdot\theta\cdot P_{ij}\cdot L_{ij}$（9）

Based on equations 4) and (5), the transportation risk on the route$\left(i,j\right)$ can be expressed as:

$TR_{ij}\text{=}\frac{4\theta\cdot Q_{ij}\cdot P_{ij}}{\pi\cdot\mu^{2}\cdot T^{2}+4\mu\cdot T\cdot H}$（10）

Transportation risk is represented by:

$f_{2}=\min\left(\sum_{i,j\in N} TR_{ij}\cdot\mu T\cdot P_{ij}\right)=\min\frac{\sum_{i,j\in N} 4\theta\cdot Q_{ij}\cdot P_{ij}\cdot\mu T}{\pi\cdot\mu^{2}\cdot T^{2}+4\mu\cdot T\cdot H}$（11）.”

3. The manuscript states that the weight ratio of optimization objectives lacks a basis. Can you clarify how you determined the weights for transportation costs and risks in your model?

According to the reviewer’s requirement, relevant statements have been added to in Section 2.

“Therefore, based on the environmental transmission characteristics of viruses, this paper proposes a multi-objective, three-dimensional risk measurement model that considers the risk of virus transmission and the generalized system cost. Moreover, this paper introduces the impact of road traffic volume on the risk of virus transmission, and constructs a model of transfer point locations - transportation routes with the goal of minimizing total system cost and transmission risk. Finally, Pareto solution is obtained based on minimum envelope clustering and NSGA-II.”

According to the reviewer’s requirement, relevant statements have been added to in Section 5.3.

“From experience, it is known that the local search strategy, genetic rules, and mutation rules of basic genetic algorithm have a significant impact on the quality and computation time of solutions when solving route problems. Therefore, this paper focuses on the shortcomings of basic genetic algorithms, such as delayed information feedback, slow search speed, and strong dependence on the initial population, made improvements in genetic coding, fitness calculation, and proposed NSGA-II.. This algorithm uses non-dominated sorting to reduce the complexity of the algorithm, and uses crowding distance and crowding distance comparison operators to ensure the diversity of the population, accelerating the convergence speed of this algorithm. And use Matlab to write the program, ultimately designing an improved NSGA-II [24-25].

(2) The selection strategy of Pareto solution

In response to the difficulty in determining the weights of indicators in traditional multi-objective optimization problems, this paper uses Pareto solution to balance the importance of multiple objectives. Pareto solution refers to a solution that cannot be improved on one objective without being inferior to other objectives under given resource conditions. In other words, if there is a change that makes at least one person better without making anyone worse off, then this change cannot further improve the solutions of other objective functions, which constitute the Pareto solution set.”

4. You mention a lack of stability and sensitivity analysis. Could you provide a plan for how you intend to conduct this analysis in future work?

According to the reviewer’s requirement, relevant statements have been added to in Section 7.

“Future research can be expanded from the following aspects: (1) increasing sensitivity analysis. In cost sensitivity analysis, adding uncertain demand to the expected number of vehicles, fuel costs under different load conditions of vehicles, and introducing the time-varying characteristics of road traffic in traffic environment sensitivity to affect transportation risks and rescue efficiency. (2) Consider the risk characteristics of different medical waste and the impact of climate conditions in different geographical regions on risk transmission. (3) In terms of algorithms, research on parallel processing of crossover and mutation operators can be strengthened to reduce the time complexity of the algorithm and improve its efficiency. At the same time, emerging technologies such as machine learning and artificial intelligence can be integrated, such as combining big data technology to analyze the impact of time-varying weather conditions and road traffic conditions on the risk of medical waste transportation, and combining deep reinforcement learning technology to optimize vehicle routes.”

5. In the NSGA-II algorithm, how is the initial population generated? What criteria are used to ensure diversity in the initial solutions?

According to the reviewer’s requirement, relevant statements have been added to in Section 5.3.

“(1) Initialize the population

The quality of initial feasible solutions will directly affect the efficiency of swarm intelligence optimization algorithms. In NSGA-II, a random generation method is used to generate the initial population, which may lead to insufficient population diversity and low convergence accuracy of the algorithm. To overcome this deficiency, this paper adopts the following improvement method to generate the initial population:

Step 1: Randomly generate a temporary population with a size of $2\times inn$, use three objective functions $f_{1},f_{2},f_{3}$ as indicators to calculate the spatial distance $w_{i}$ between each chromosome and other chromosomes, and sort $w_{i}$ accordingly.

$w_{i}=\sum_{j,i\neq j} (|f_{1}^{i}-f_{1}^{j}|+|f_{2}^{i}-f_{2}^{j}|+|f_{3}^{i}-f_{3}^{j}|)$（31）

Step 2: Select $inn$ chromosomes from $w_{i}$ in descending order to form the initial population.

(4) Crowding Distance

In NSGA-II, crowding distance is an important criterion for evaluating individual strengths and weaknesses. Crowding distance=(individual fitness - average fitness)/standard deviation. Under the same level of non dominated sorting, calculate the sum of the relative distances between a chromosome and its adjacent points in space at multiple objective function values. Crowding distance can ensure that the algorithm converges to a relatively uniform Pareto domain, achieving the goal of maintaining population diversity.”

6. W Ensure that all references are up-to-date and formatted according to the journal's guidelines. Some citations appear to be missing or incomplete. In general, the authors are recommended to refer to more recent papers in the manuscript, such as: doi.org/10.1016/j.eswa.2023.121035, doi.org/10.1109/TITS.2023.3285430.

According to the reviewer’s requirement, relevant statements have been added to in Section 2.

“Wang Nengmin et al. [20] defined the infection process and the transmission risk of infected populations, set up multiple disposal centers, developed a dual objective route model for medical waste collection to optimize infection risk and transportation costs, and obtained Pareto solution by combining weighted economic constraint method.”

7. The case study in Yi-bin City is mentioned, but details on the data used (e.g., waste generation rates, vehicle capacities) are sparse.

According to the reviewer’s requirement, relevant statements have been added to in Section 6.1.

“In addition, transportation risks have many uncertainties. To simplify the model in this paper, the following assumptions are made: the annual average wind speed in Yibin City is 4.43km/h. Probability of waste being infectious is 0.6; The population density of all lines in the region is 7500 person/km^2^; The vehicle height is 1 meter; The rescue capacity of the rescue center is 1000kg/h. Driving speed $v=40km/h$, vehicle load capacity in first echelon is 5t, fixed cost is 500 yuan, unit mileage transportation cost of 10 yuan, vehicle load capacity in second echelon is 1t, fixed cost is 200 yuan, unit mileage transportation cost is 5 yuan. ”

8. What assumptions did you make in your model regarding vehicle capacities, waste generation rates, and transit point locations? How might these assumptions impact the results?

According to the reviewer’s requirement, relevant statements have been added to in Section 4.2.

“(1) There is one medical waste disposal center, multiple transfer points, and multiple producing points in the area, and the disposal center is equipped with different types of vehicles to meet the transportation needs;

(2) Each producing point can only be served by one transport vehicle, and the amount of medical waste generated is less than the load capacity of the transport vehicle;”

9. Some figures and tables lack clear explanations in the text. Can you ensure that all visual aids are adequately referenced and explained?

According to the reviewer’s requirement, relevant statements have been added to in Section 6.2.

“By using Matlab to solve the problem, the average distance of each Pareto optimal solution tends to stabilize after about 160 iterations, and the Pareto front is shown in Figure 6. According to the selection strategy of Pareto solution, The recycling path of medical waste is shown in Figure 7. Among them, the total mileage of the vehicle is 92.62+18.99km, with a total cost of 1063+690 yuan and transport risk of 73.12 t·person/km^3^, requiring 3+1 vehicles (data before and after “+” are for the first and second echelons respectively).”

According to the reviewer’s requirement, relevant statements have been added to in Section 6.3.

“By using Matlab to solve the problem, the average distance of each Pareto optimal solution tends to stabilize after about 170 iterations, and the Pareto front is shown in Figure 8. The medical waste transport route of Pareto solution is shown in Figure 9. At this time, the total operating cost is 3249+1809 yuan, the transportation risk is 517.26 tons·person/km^3^, and the total mileage of vehicles is 89.78+30.87km, requiring 14+3 vehicles. The data before and after "+" represent the first and second stages, respectively.”

10. What are the limitations of your study, and how might they affect the applicability of your model in different contexts or regions?

According to the reviewer’s requirement, relevant statements have been added to in Section 7.

“However, this paper also has limitations, such as the lack of specialized qualifications and facility equipment requirements for the treatment of infectious medical waste in the study, and the possibility of irregular transportation strategies for transfer points that generate less medical waste. At the same time, this article did not consider the chemical and risk characteristics of different types of medical waste, and urban medical waste needs to be classified and treated according to different characteristics.”

11. What are the next steps for this research? Are there plans to extend the model to include other types of medical waste or different geographical areas?

According to the reviewer’s requirement, relevant statements have been added to in Section 7.

“Future research can be expanded from the following aspects: (1) increasing sensitivity analysis. In cost sensitivity analysis, adding uncertain demand to the expected number of vehicles, fuel costs under different load conditions of vehicles, and introducing the time-varying characteristics of road traffic in traffic environment sensitivity to affect transportation risks and rescue efficiency. (2) Consider the risk characteristics of different medical waste and the impact of climate conditions in different geographical regions on risk transmission. (3) In terms of algorithms, research on parallel processing of crossover and mutation operators can be strengthened to reduce the time complexity of the algorithm and improve its efficiency. At the same time, emerging technologies such as machine learning and artificial intelligence can be integrated, such as combining big data technology to analyze the impact of time-varying weather conditions and road traffic conditions on the risk of medical waste transportation, and combining deep reinforcement learning technology to optimize vehicle routes.”

**Reviewer 2 Comments to the Author**

Comments to Author:

1. I recommend the author to change the description of objective f_3 from section 4.4 to 4.2.3. It very hard and find where is the description of objective 3. Because the 4.1.1 and 4.2. is describe about f_1 and f_2.

According to the reviewer’s requirement, we change the description of objective f_3 from section 4.4 to 4.3.3

**4.3.3 Rescue model in case of infectious medical waste leakage**

When medical waste belongs to highly contagious, widely contagious, and rapidly contagious infectious substances, m is the location of infectious waste leakage, $m\in E_{ij}$. At this point, add a new objective function $f_{3}$: the fastest rescue speed, i.e. the shortest rescue distance:

$\min f_{3}=\max\left\{\frac{L_{Di}+L_{im}\cdot\beta_{ij}}{v_{D}},\frac{L_{Dj}+L_{jm}\cdot\beta_{ij}}{v_{D}}\left|Q_{ij}\neq0\right.\right\}$（12）

2. This paper use NGSA-II to find the 3 objectives optimization problem. However, I cannot see you Pareto solutions of your problem. I recommend the author to added the figure of Pareto solutions in your manuscript.

According to the reviewer’s requirement, relevant statements have been added to in Section 6.2.

By using Matlab to solve the problem, the average distance of each Pareto optimal solution tends to stabilize after about 160 iterations, and the Pareto front is shown in Figure 6. According to the selection strategy of Pareto solution, The recycling path of medical waste is shown in Figure 7. Among them, the total mileage of the vehicle is 92.62+18.99km, with a total cost of 1063+690 yuan and transport risk of 73.12 t·person/km^3^, requiring 3+1 vehicles (data before and after “+” are for the first and second echelons respectively).

Figure6. Solution obtained by NSGA-II (Instance #1)

According to the reviewer’s requirement, relevant statements have been added to in Section 6.3.

By using Matlab to solve the problem, the average distance of each Pareto optimal solution tends to stabilize after about 170 iterations, and the Pareto front is shown in Figure 8. The medical waste transport route of Pareto solution is shown in Figure 9. At this time, the total operating cost is 3249+1809 yuan, the transportation risk is 517.26 tons·person/km^3^, and the total mileage of vehicles is 89.78+30.87km, requiring 14+3 vehicles. The data before and after "+" represent the first and second stages, respectively.

Figure8. Solution obtained by NSGA-II (Instance #2)

3. Literature review is weak, few recent year literature papers are reviewed and included in the literature section. Need to add more number of recent year literature papers.

According to the reviewer’s requirement, relevant statements have been added to in Section 5.3.

The selection strategy for Pareto solutions is as follows:

① If there is a unique scheme $i\in U^{*}$ that satisfies the minimum $\left|risk\left(i\right)-\overline{risk}\right|$, then plan$i$ is chosen as the Pareto solution.

② If there is scheme $i,j\in U^{*}$ that satisfies the minimum $\left|risk\left(i\right)-\overline{risk}\right|\text{=}\left|risk\left(j\right)-\overline{risk}\right|$ and $\left|risk\left(i\right)-\overline{risk}\right|$, $\left|cost\left(i\right)-\overline{cost}\right|\text{<}\left|cost\left(j\right)-\overline{cost}\right|$, then plan$i$ is chosen as the Pareto solution.

③If there is scheme $i,j\in U^{*}$ that satisfies the minimum $\left|risk\left(i\right)-\overline{risk}\right|\text{=}\left|risk\left(j\right)-\overline{risk}\right|$ and $\left|risk\left(i\right)-\overline{risk}\right|$, $\left|cost\left(i\right)-\overline{cost}\right|\text{=}\left|cost\left(j\right)-\overline{cost}\right|$, then when $risk\left(i\right)<risk\left(j\right)$, plan$i$ is chosen as the Pareto solution.

4. In general, the history search of multi/many-objective optimization can measured by multi-objective optimization metrics. I recommend the author to use some metrics to be shown your optimum solution has been converged.

According to the reviewer’s requirement, relevant statements have been added to in Section 6.2 and 6.3.

By using Matlab to solve the problem, the average distance of each Pareto optimal solution tends to stabilize after about 160 iterations, and the Pareto front is shown in Figure 6. According to the selection strategy of Pareto solution, The recycling path of medical waste is shown in Figure 7. Among them, the total mileage of the vehicle is 92.62+18.99km, with a total cost of 1063+690 yuan and transport risk of 73.12 t·person/km^3^, requiring 3+1 vehicles (data before and after “+” are for the first and second echelons respectively).

By using Matlab to solve the problem, the average distance of each Pareto optimal solution tends to stabilize after about 170 iterations, and the Pareto front is shown in Figure 8. The medical waste transport route of Pareto solution is shown in Figure 9. At this time, the total operating cost is 3249+1809 yuan, the transportation risk is 517.26 tons·person/km^3^, and the total mileage of vehicles is 89.78+30.87km, requiring 14+3 vehicles. The data before and after "+" represent the first and second stages, respectively.

5. This paper try to compared the results of improve NSGA-II and traditional NSGA-II. However, the comparison isn't fair because now you due with the multi-objective optimization. I recommend the author compared the Pareto solution from each method and compared the convergence rate of each algorithm using some metric such as hyper volume.

According to the reviewer’s requirement, relevant statements have been added to in Section 6.4.

In addition, we calculated the hyper volumes of each result, as shown in Figure 10. Hyper volume is an important parameter reflecting the Pareto front quality. It can be seen that the improved NSGA-II has the highest hyper volume in both of the previous examples, and its results are better than those of the basic genetic algorithm.

Figure10. Hyper volumes of each result

We tried our best to improve the manuscript and made some changes in the manuscript. These changes will not influence the content and framework of the paper. And here we marked in yellow in revised paper. We appreciate for Editors/Reviewers’ warm work earnestly, and hope that the correction will meet with approval.

Once again, thank you very much for your comments and suggestions.
