# Supplementary material for: Research on optimization of transportation routes for infectious medical waste
Source: PLoS One. 2025 Sep 26;20(9):e0330996. doi: 10.1371/journal.pone.0330996 (PMC12469087; doi:10.1371/journal.pone.0330996)
Supplement: Attachment — Submitted filename: Response720.docx [file pone.0330996.s019.docx]

**Original Manuscript ID:** PONE-D-24-35149R1

**Original Article Title: “Research on Optimization of Transportation Routes for Infectious Medical Waste”**

**To:** Plos One Editor

**Re:** Response to reviewers

Dear Editor,

Thank you for allowing a resubmission of our manuscript, with an opportunity to address the reviewers’ comments.

We are uploading (a) our point-by-point response to the comments (below) (response to reviewers), (b) an updated manuscript with yellow highlighting indicating changes, and (c) a clean updated manuscript without highlights (Word main document).

Best regards,

Hao Chen, Libo Li, Xuerui Qin*, Wenxian Wang.

**Reviewer 3 Comments to the Author**

Comments to Author:

1. The abstract would benefit from a clearer emphasis on the main numerical findings of the study. Explicitly stating quantitative results will strengthen the perceived value of the work.

**According to the reviewer’s requirement,** we rewrite the **abstract** as follows.

‘During the pandemic, the amount of infectious medical waste has increased dramatically. Currently, the medical waste recycling process generally suffers from defects such as long distances, high costs, and a lack of emergency response mechanisms. This paper addresses the problem of medical waste collection and route optimization for regions with multiple vehicle types and stages. It comprehensively considers factors such as transportation costs, distance, vehicle allocation, and contamination risks during the collection and distribution of medical waste. The goal is to minimize transportation costs and risks, with constraints including uniqueness, connectivity between nodes, and vehicle load capacity. A segmented collection approach is used to model the medical waste collection process. An optimization method for medical waste collection site selection and vehicle routing is proposed. Given the NP-hard nature of the problem, a location allocation method based on minimum envelope clustering analysis is employed, and an improved NSGA-II algorithm incorporating a fast non-dominated sorting mechanism is designed to obtain Pareto optimal solutions. Comparing with the results of traditional genetic algorithms through simulation, the results show that using the improved NSGA-II to solve practical problems: 1. When the production of medical waste is flat (1 disposal center, 4 backup transfer points, 58 producing points), the total cost is reduced by 13.94%, the total mileage is reduced by 7.17%, the full load rate is increased by 6.14%, and the convergence time is 26 seconds. 2. When the production of medical waste increased significantly (1 disposal center, multiple backup transfer points, 58 producing points), the total cost, total mileage, and transportation risk were reduced by 9.50%, 10.35%, and 2.03%, respectively, and the full load rate increased by 5.98%. The final results also indicate that compared to the results obtained by traditional genetic algorithms, the improved NSGA-II algorithm performs better in solving the optimization problem of infectious medical waste transportation routes. ’

2. The introduction should avoid vague qualifiers such as "important issue" and instead provide quantified evidence, preferably supported by current references or statistics. Additionally, the articulation of research gaps needs to be more prominent. The authors are encouraged to use visual or structural enhancements—such as bullet points or bolded text—to emphasize the novelty and motivation of their work.

**According to the reviewer’s requirement,** relevant statements have been added to in Section **Introduction**.

‘The management and recycling of medical waste has gradually become one of the important environmental, health, and social issues. Due to population growth and urbanization, especially the increase in the number of COVID-19 and other epidemics, the amount of medical waste is also continuously increasing, and the disposal of medical waste has become a focus [1].’

|  | Generated（kiloton） | Processed（kiloton） |
| --- | --- | --- |
| 2015 | 1882 | 1054 |
| 2016 | 1958 | 1114.6 |
| 2017 | 2090 | 1216.3 |
| 2018 | 2200 | 1318 |
| 2019 | 2290 | 1374.5 |
| 2020 | 2410 | 1487.5 |
| 2021 | 2520 | 1544 |
| 2022 | 2636 | 1654 |

Figure1.The amount of medical waste generated and processed in China

3. The literature review section identifies several relevant studies, but does so without critical analysis. It is recommended that the authors include a summary table comparing key features of the most closely related works with their own proposed approach. This will help clarify the distinct contributions of the current study.

**According to the reviewer’s requirement,** relevant statements have been added to in Section **Literature Review**.

‘Table 1 provides a detailed comparison between current research and previous studies on optimizing vehicle delivery routes. From the existing research results, scholars at home and abroad have conducted a lot of research on waste site selection and path optimization, and there is also sufficient research on the site selection and path optimization of hazardous waste disposal centers. However, although medical waste is also classified as hazardous waste, there are still differences in essence. On the one hand, hospitals need to be considered in the disposal process of medical waste, and on the other hand, the disposal center for infectious waste requires a dedicated disposal center, which is different from other waste disposal centers. Therefore, the research on site selection and path optimization of medical waste recycling centers is different from other waste disposal methods in practical situations. At present, there is a lack of quantitative research literature on the site selection and path optimization of medical waste disposal, and there are more qualitative studies. Especially, few scholars have paid attention to the more detailed field of infectious medical waste disposal. ’

Tab.1 Comparison of research contributions with existing studies

| **Authors** | **Cost** | **Capacity** | **Risk** | **Mileage** | **Objective** | **Method** | **Environment** |
| --- | --- | --- | --- | --- | --- | --- | --- |
| **Stancu (1976)** | **√** | √ |  |  | Multi | MILP | Deterministic‌ |
| **Stern (1981)** |  |  |  | √ | Single | MILP | Deterministic‌ |
| **Listers (2005)** | √ |  |  |  | Single | MILP | Stochastic |
| **Aylin (2008)** | √ |  |  |  | Single | MILP | Stochastic |
| **Nie (2018)** | √ | √ |  |  | Multi | GA | Deterministic‌ |
| **Dong (2022)** | √ |  | √ |  | Multi | GA | Deterministic‌ |
| **Shi (2011)** |  |  |  | √ | Single | ACS | Deterministic‌ |
| **He (2007)** | √ |  | √ |  | Multi | Greedy | Deterministic‌ |
| **Osaba (2018)** | √ |  |  | √ | Multi | Bat | Deterministic‌ |
| **Le (2016)** | √ |  |  | √ | Multi | MILP | Deterministic‌ |
| **Liu (2016)** |  |  |  | √ | Single | Neighbor | Deterministic‌ |
| **Ma (2016)** | √ |  |  | √ | Multi | MILP | Deterministic‌ |
| **Alsh (2017)** | √ |  |  | √ | Multi | MILP | Stochastic |
| **Wang (2023)** | √ |  | √ |  | Multi | GA | Deterministic‌ |
| **This article** | √ | √ | √ | √ | Multi | NSGA-II | Deterministic‌ |

4. In the methodology section, it is important to justify the choice of the improved NSGA-II over other leading multi-objective evolutionary algorithms (MOEAs). A comparative rationale, perhaps supported by relevant citations or empirical evidence, would enhance the credibility of the algorithmic selection.

**According to the reviewer’s requirement,** relevant statements have been added to in Section **Solution Algorithm.**

‘This paper models the location and routing optimization of infectious medical waste recycling, which leads to an explosive increase in computational complexity as the number of nodes grows, causing the calculations to be plagued by dimensional issues. Additionally, the model incorporates time windows, damage rates, and time-varying transportation speeds, containing many variables and constraints, resulting in a large-scale, multi-objective optimization problem. To address this, a heuristic search algorithm suitable for solving this model is proposed.

The NSGA-II algorithm inherits the "survival of the fittest" group search mechanism from basic genetic algorithms, offering good parallel computing and global search capabilities. Furthermore, the algorithm introduces an elitism strategy to ensure that certain high-quality individuals in the population are not discarded during the evolution process. By using a crowding distance and a crowding comparison operator, it overcomes the limitation of traditional multi-objective algorithms, which require manually setting the objective weights. This ensures that the individuals in the approximate Pareto domain are evenly distributed across the entire Pareto domain, thereby maintaining the diversity of the population.

Based on this, considering the uncertainty of transfer center locations in the research problem, which can lead to a large number of nodes and excessively long solving time and computational load, a two-stage solving strategy is designed. The first stage involves a location-allocation algorithm based on minimum enclosing clustering analysis, while the second stage uses an improved NSGA-II algorithm for routing optimization. As a result, the problem in this paper is transformed into a 2E-MOLRP problem (two-echelon multi-objective localization path problem), which further reduces the complexity of solving the problem.’

5. Figures should be more clearly referenced and interpreted within the main text. Furthermore, the use of units and performance metrics should be standardized and clearly defined throughout the experimental analysis.

**According to the reviewer’s requirement**, relevant statements have been added to in Section **6.1**.

‘Due to the significant increase in the amount of infectious medical waste during the COVID-19 pandemic, the volume of such waste is expected to rise substantially, leading to the capacity of existing transfer centers being unable to meet the demand of the regional system. As a result, additional transfer centers need to be established. Meanwhile, due to the sharp rise in the volume and frequency of infectious medical waste transportation, it is necessary to deploy a certain number of rescue centers within the region to ensure that the coverage area of the rescue centers fully encompasses the Lingang University Town area. Furthermore, given the uncertainty in the measurement of transportation risks, the remaining relevant parameters are set as shown in Table 4 to simplify the model in this paper. ’

**Tab.4 Explanation and Values of Relevant Parameters in the Case**

| **Symbols** | **Parameter** | **Values** |
| --- | --- | --- |
| $\mu$ | Average wind speed | 4.43km/h |
| $\theta$ | Probability of waste being infectious | 0.6 |
| $P_{ij}$ | The population density of all lines in the region | 7500per/km^2^ |
| $H$ | Vehicle height | 1 meter |
| $FC_{g}$ | Cost of adding rescue centers | 50 million yuan |
| $F_{D}$ | The rescue capacity of the rescue center | 1000kg/h |
| $R_{D}$ | The coverage radius of the rescue center | 500 meters |
| $v_{D}$ | Driving speed | 40 km/h |
| $Z_{k,1}$ | Vehicle load capacity in first echelon | 5 t |
| $Z_{k,2}$ | Vehicle load capacity in second echelon | 1 t |
| $FC_{k,1}$ | Fixed cost in first echelon | 5million yuan |
| $FC_{k,2}$ | Fixed cost in second echelon | 2million yuan |
| $fc_{k,1}$ | Unit mileage transportation costin first echelon | 100 thousand yuan |
| $fc_{k,2}$ | Unit mileage transportation costin second echelon | 50 thousand yuan |

6. The limitations section is appreciated and a good starting point. However, it would benefit from further elaboration to include:

• Real-time traffic uncertainty;

• Dynamic generation rates of medical waste;

• Regulatory constraints and compliance issues related to infectious waste logistics.

**According to the reviewer’s requirement**, relevant statements have been added to in Section **Conclusions**.

‘(1) increasing sensitivity analysis. In cost sensitivity analysis, adding uncertain demand to the expected number of vehicles, fuel costs under different load conditions of vehicles, and introducing the time-varying characteristics of road traffic in traffic environment sensitivity to affect transportation risks and rescue efficiency. (2) Increasing the uncertainty of urban road traffic and its impact on the travel paths of medical waste recycling vehicles, such as considering the dynamic changes in real-time transportation conditions of urban roads in the calculation of rescue speed objectives. (3) Including the dynamic generation rate of medical waste and its impact on cost objectives, such as factoring in the vehicle idle cost coefficient under different medical waste generation rates in the fixed vehicle usage costs. (4) Considering the risk characteristics of different types of medical waste and the impact of climate conditions in different geographical areas on the spread of risks, as well as regulatory constraints and compliance issues related to infectious waste logistics. Additionally, extending the model to include other types of medical waste or different geographical regions.’

**Reviewer 4 Comments to the Author**

My comments are as follows:

1. In the introduction, you need to connect the state of the art to your paper goals. Please follow the literature review by a clear and concise state of the art analysis. This should clearly show the knowledge gaps identified and link them to your paper goals. Please reason both the novelty and the relevance of your paper goals. Clearly discuss what the previous studies that you are referring to. What are the Research Gaps/Contributions? Please note that the paper may not be considered further without a clear research gap and novelty of the study and compare your paper with municipal cooperative waste supply chain papers and sustainable medical waste supply chain network.

**According to the reviewer’s requirement**, relevant statements have been added to in **Introduce** and **Literature Review**.

‘The design of a medical waste recycling system refers to the process where, within a certain research scope, all nodes where medical waste is generated are collected by professional recycling vehicles to disposal points under the constraint of vehicle load capacity. Therefore, the key to designing a medical waste recycling system lies in establishing an appropriate number and location of medical waste transfer centers, and then determining the transportation routes for all vehicles involved in recycling, so that the overall cost of the waste recycling plan is minimized. At the same time, the operational routes of the recycling vehicles should minimize the risk of infection spread to surrounding residents. It can be seen that the core issue of the medical waste recycling system design addressed in this paper is the integrated optimization of the selection of recycling centers and vehicle routing. An important premise for the successful implementation of this method is that basic data such as the locations of medical waste generation nodes, the number of delivery vehicles, their load capacity, the infection range, and the number of residents within the infection zone are given values. The main contribution of this paper is reflected in:

(1) During the period when a large amount of medical waste is produced, the recycling process involves multiple vehicles, high costs, and high infectious risks. Therefore, this paper considers the selection of medical waste transfer points and transportation route optimization under the risk of infection, making the recycling plan more in line with the actual situation.

(2) This paper establishes a multi-objective 0-1 decision integer programming model for the selection of medical waste transfer points and the optimization of transportation routes, with transportation costs and infection risks as optimization objectives.

(3) This paper proposes an improved NSGA-II, which proves its strong applicability in model solving by taking Yibin City during the COVID-19 as an example, and has certain advantages compared to traditional genetic algorithms.’

‘‘Table 1 provides a detailed comparison between current research and previous studies on optimizing vehicle delivery routes. From the existing research results, scholars at home and abroad have conducted a lot of research on waste site selection and path optimization, and there is also sufficient research on the site selection and path optimization of hazardous waste disposal centers. However, although medical waste is also classified as hazardous waste, there are still differences in essence. On the one hand, hospitals need to be considered in the disposal process of medical waste, and on the other hand, the disposal center for infectious waste requires a dedicated disposal center, which is different from other waste disposal centers. Therefore, the research on site selection and path optimization of medical waste recycling centers is different from other waste disposal methods in practical situations. At present, there is a lack of quantitative research literature on the site selection and path optimization of medical waste disposal, and there are more qualitative studies. Especially, few scholars have paid attention to the more detailed field of infectious medical waste disposal. ’

Tab.1 Comparison of research contributions with existing studies

| **Authors** | **Cost** | **Capacity** | **Risk** | **Mileage** | **Objective** | **Method** | **Environment** |
| --- | --- | --- | --- | --- | --- | --- | --- |
| **Stancu (1976)** | **√** | √ |  |  | Multi | MILP | Deterministic‌ |
| **Stern (1981)** |  |  |  | √ | Single | MILP | Deterministic‌ |
| **Listers (2005)** | √ |  |  |  | Single | MILP | Stochastic |
| **Aylin (2008)** | √ |  |  |  | Single | MILP | Stochastic |
| **Nie (2018)** | √ | √ |  |  | Multi | GA | Deterministic‌ |
| **Dong (2022)** | √ |  | √ |  | Multi | GA | Deterministic‌ |
| **Shi (2011)** |  |  |  | √ | Single | ACS | Deterministic‌ |
| **He (2007)** | √ |  | √ |  | Multi | Greedy | Deterministic‌ |
| **Osaba (2018)** | √ |  |  | √ | Multi | Bat | Deterministic‌ |
| **Le (2016)** | √ |  |  | √ | Multi | MILP | Deterministic‌ |
| **Liu (2016)** |  |  |  | √ | Single | Neighbor | Deterministic‌ |
| **Ma (2016)** | √ |  |  | √ | Multi | MILP | Deterministic‌ |
| **Alsh (2017)** | √ |  |  | √ | Multi | MILP | Stochastic |
| **Wang (2023)** | √ |  | √ |  | Multi | GA | Deterministic‌ |
| **This article** | √ | √ | √ | √ | Multi | NSGA-II | Deterministic‌ |

2. Constraints such as “uniqueness,” “logicality,” and “load capacity” are vaguely defined. Can you explicitly formulate these constraints mathematically within the model and explain their operational relevance?

**According to the reviewer’s requirement**, relevant statements have been added to in Section **4.4**.

(1) Uniqueness constraint:

The uniqueness constraint of the model includes the following two aspects: Firstly, for any medical institution node, there is only one transport vehicle to transport it, as shown in formula (13); Secondly, vehicles departing from the transfer point to collect waste at medical institutions and ultimately returning to the corresponding transfer point，As shown in formulas (14) and (15).Vehicles that depart from the disposal center to load waste at the transfer point and ultimately return to the corresponding disposal center，As shown in formulas (16) and (17):

$\sum_{i,j\in V} \sum_{k\in K} \left(\beta_{ij}\cdot\gamma_{k}\right)=1$（13）

Vehicles departing from the transfer point to collect waste at medical institutions and ultimately returning to the corresponding transfer point:

$\sum_{i\in V} \sum_{g\in G} \sum_{k\in K} \left(\alpha_{g}\cdot\beta_{gi}\cdot\gamma_{k}\right)=1$（14）

$\sum_{j\in V} \sum_{g\in G} \sum_{k\in K} \left(\alpha_{g}\cdot\beta_{jg}\cdot\gamma_{k}\right)=1$（15）

Vehicles that depart from the disposal center to load waste at the transfer point and ultimately return to the corresponding disposal center:

$\sum_{g\in G} \sum_{s\in S} \sum_{k\in K} \left(\alpha_{g}\cdot\beta_{sg}\cdot\gamma_{k}\right)=1$（16）

$\sum_{g'\in G} \sum_{s\in S} \sum_{k\in K} \left(\alpha_{g'}\cdot\beta_{g's}\cdot\gamma_{k}\right)=1$（17）

(2) Logical constraints:

Connectivity constraints between nodes. The inter node connectivity constraint refers to the requirement that any two nodes should have connectivity, as shown in formula (18). There is no mutually ineffective transportation between each transfer center and each processing center, as shown in formula (19). For any medical institution, there is only one vehicle that comes to serve, and at the same time, only this vehicle leaves the medical institution, as shown in formula (20).

$\sum_{i,j,g,s\in N} \sum_{k\in K} \alpha_{g}\cdot\beta_{ij}\cdot\gamma_{k}-\sum_{i,j,g,s\in N} \sum_{k\in K} \alpha_{g}\cdot\beta_{ji}\cdot\gamma_{k}=0$（18）

No invalid transportation shall be carried out between each transfer point and each processing center:

$\sum_{g,g'\in N} \beta_{gg'}=\sum_{s,s'\in N} \beta_{ss'}=0$（19）

For any medical institution with only one vehicle to collect waste and only one vehicle to leave the institution:

$\sum_{i,j\in V} \sum_{g\in G} \sum_{k\in K} \beta_{gi}\cdot\gamma_{k}+\sum_{i,j\in V} \sum_{k\in K} \beta_{ji}\cdot\gamma_{k}=1$（20）

$\sum_{i,j\in V} \sum_{k\in K} \beta_{ij}\cdot\gamma_{k}+\sum_{i,j\in V} \sum_{g\in G} \sum_{k\in K} \beta_{ig}\cdot\gamma_{k}=1$（21）

(3) Capacity constraints:

Conservation of weight of medical waste on transportation routes:

$\sum_{i,j,n\in N} \sum_{k\in K} Q_{ij}\cdot\gamma_{k}+q_{j}=\sum_{i,j,n\in N} \sum_{k\in K} Q_{jn}\cdot\gamma_{k}$（22）

The total weight of medical waste shall not exceed the maximum load capacity of the vehicle. At transfer points and disposal centers, the total weight of medical waste does not exceed the capacity limit:

$\sum_{i,j\in V} \sum_{k\in K} \beta_{ij}\cdot\gamma_{k}\cdot q_{j}\leq Z_{k}$（23）

$\sum_{g\in G} \alpha_{g}\beta_{jg}\left(\sum_{i,j\in V} \sum_{k\in K} \beta_{ij}\cdot\gamma_{k}\cdot q_{i}\right)\leq Z_{g}$（24）

$\sum_{s\in S} \sum_{g\in G} \alpha_{g}\beta_{jg}\beta_{gs}\left(\sum_{i,j\in V} \sum_{k\in K} \beta_{ij}\cdot\gamma_{k}\cdot q_{i}\right)\leq Z_{s}$（25）

At the same time, it is necessary to add constraints that the amount of leaked infectious medical waste cannot exceed the processing capacity of the rescue center:

$\sum_{i,j\in V} \sum_{k\in K} \beta_{ij}\cdot q_{i}\cdot\gamma_{k}\leq F_{D}$（26）

(4)Limitations on the scope and quantity of rescue centers:

All nodes and routes are within the rescue range of a certain rescue center:

$d_{Dij}\leq R_{D}$（27）

The number of rescue centers is greater than or equal to 1:

$\sum D\geq1$（28）

3. The paper states it adopts a "segmented strategy" for modeling without adequately explaining what this segmentation entails or how it improves over a holistic model. Could you elaborate on what stages are modeled and how segmentation affects the computational efficiency or route design?

**According to the reviewer’s requirement,** relevant statements have been added to in Section **Solution Algorithm.**

‘This paper models the location and routing optimization of infectious medical waste recycling, which leads to an explosive increase in computational complexity as the number of nodes grows, causing the calculations to be plagued by dimensional issues. Additionally, the model incorporates time windows, damage rates, and time-varying transportation speeds, containing many variables and constraints, resulting in a large-scale, multi-objective optimization problem. To address this, a heuristic search algorithm suitable for solving this model is proposed.

The NSGA-II algorithm inherits the "survival of the fittest" group search mechanism from basic genetic algorithms, offering good parallel computing and global search capabilities. Furthermore, the algorithm introduces an elitism strategy to ensure that certain high-quality individuals in the population are not discarded during the evolution process. By using a crowding distance and a crowding comparison operator, it overcomes the limitation of traditional multi-objective algorithms, which require manually setting the objective weights. This ensures that the individuals in the approximate Pareto domain are evenly distributed across the entire Pareto domain, thereby maintaining the diversity of the population.

Based on this, considering the uncertainty of transfer center locations in the research problem, which can lead to a large number of nodes and excessively long solving time and computational load, a two-stage solving strategy is designed. The first stage involves a location-allocation algorithm based on minimum enclosing clustering analysis, while the second stage uses an improved NSGA-II algorithm for routing optimization. As a result, the problem in this paper is transformed into a 2E-MOLRP problem (two-echelon multi-objective localization path problem), which further reduces the complexity of solving the problem.’

4. While the improved NSGA-II is claimed to outperform a traditional genetic algorithm, no formal statistical validation or benchmark comparison is shown. Could the authors compare with at least one other modern multi-objective algorithm (e.g., MOEA/D, SPEA2) and report metrics such as hypervolume or spread?

**According to the reviewer’s requirement**, relevant statements have been added to in Section **6.4**.

‘To further validate the performance of the improved NSGA-II algorithm and the model, a comparison was made between the improved NSGA-II algorithm and the traditional genetic algorithm. The initial population, genetic probability, mutation probability, and termination algebra parameters of each system are set the same. At the same time, considering the actual situation during the period, the amount of medical waste produced will fluctuate with government policies, the spread of the epidemic, and other factors. The plans will be operated separately under uncertain demand conditions, and the comparison of the results is shown in Table 7.’

**Table 7. Comparison of calculation results**

|  | Improved NSGA-II | | MOEA/D | | Traditional genetic algorithm | | Average optimization rate |
| --- | --- | --- | --- | --- | --- | --- | --- |
| Objective function | low waste production | increased waste production | low waste production | increased waste production | low waste production | increased waste production |  |
| The amount of medical waste produced(kg) | 2209 | 13435 | 2209 | 13435 | 2209 | 13435 | / |
| Vehicle driving distance(km) | 111.61 | 120.65 | 111.61 | 130.24 | 120.23 | 134.58 | 8.76% |
| Number of vehicles | 4 | 17 | 4 | 18 | 5 | 19 | 12.78% |
| Total transportation cost(yuan) | 1753 | 5058 | 1753 | 5303 | 2037 | 5589 | 11.72% |
| Transportation risk(t·person/km^3^) | - | 517.26 | - | 520.58 | - | 527.97 | 2.03% |
| The full load rate of the vehicle | 55.23% | 92.66% | 55.23% | 90.12% | 49.09% | 86.68% | 6.06% |

‘In addition, we calculated the hyper volumes of each result, as shown in Figure 10. Hyper volume is an important parameter reflecting the Pareto front quality. It can be seen that the improved NSGA-II has the highest hyper volume in both of the previous examples, and its results are better than those of the basic genetic algorithm.’

Figure10. Hypervolumes of each result

5. The problem instances and solution parameters (e.g., 58 production points, locations, transfer point limits) are not fully detailed. Can the authors provide a table summarizing the data and parameters used in simulations to ensure replicability?

**According to the reviewer’s requirement**, relevant statements have been added to in Section **6.1**.

‘Due to the significant increase in the amount of infectious medical waste during the COVID-19 pandemic, the volume of such waste is expected to rise substantially, leading to the capacity of existing transfer centers being unable to meet the demand of the regional system. As a result, additional transfer centers need to be established. Meanwhile, due to the sharp rise in the volume and frequency of infectious medical waste transportation, it is necessary to deploy a certain number of rescue centers within the region to ensure that the coverage area of the rescue centers fully encompasses the Lingang University Town area. Furthermore, given the uncertainty in the measurement of transportation risks, the remaining relevant parameters are set as shown in Table 4 to simplify the model in this paper. ’

**Tab.4 Explanation and Values of Relevant Parameters in the Case**

| **Symbols** | **Parameter** | **Values** |
| --- | --- | --- |
| $\mu$ | Average wind speed | 4.43km/h |
| $\theta$ | Probability of waste being infectious | 0.6 |
| $P_{ij}$ | The population density of all lines in the region | 7500per/km^2^ |
| $H$ | Vehicle height | 1 meter |
| $FC_{g}$ | Cost of adding rescue centers | 50 million yuan |
| $F_{D}$ | The rescue capacity of the rescue center | 1000kg/h |
| $R_{D}$ | The coverage radius of the rescue center | 500 meters |
| $v_{D}$ | Driving speed | 40 km/h |
| $Z_{k,1}$ | Vehicle load capacity in first echelon | 5 t |
| $Z_{k,2}$ | Vehicle load capacity in second echelon | 1 t |
| $FC_{k,1}$ | Fixed cost in first echelon | 5million yuan |
| $FC_{k,2}$ | Fixed cost in second echelon | 2million yuan |
| $fc_{k,1}$ | Unit mileage transportation costin first echelon | 100 thousand yuan |
| $fc_{k,2}$ | Unit mileage transportation costin second echelon | 50 thousand yuan |

6. The transport risk is quantified as “t·person/km³,” which is unclear and lacks explanation. What is the theoretical or empirical basis for this risk metric, and how does it influence decision-making in route optimization?

**According to the reviewer’s requirement**, relevant statements have been added to in Section **4.3.2**.

The total transportation risk is represented by the product of transportation risk, diffusion time, and population density along each section of the route.

The radius of the diffusion range is:

$R\text{=}\mu\cdot T$（5）

Among them, the diffusion time $T$can be expressed as:

$T\text{=}\frac{d_{Dij}}{v_{D}}+\frac{Q_{ij}}{F_{D}}$（6）

The cross-sectional area of the diffusion range is:

$S\text{=}\frac{1}{2}\pi R^{2}+2R\cdot H$（7）

$L_{ij}$ is the length of the route$\left(i,j\right)$, and the volume of diffusion range can be expressed as:

$O_{ij}\text{=}S\cdot L_{ij}=\left(\frac{1}{2}\pi R^{2}+2R\cdot H\right)\cdot L_{ij}$（8）

The concentration of the virus is represented by the population within the current diffusion range:

$M=\pi\cdot\mu^{2}\cdot T^{2}\cdot\theta\cdot P_{ij}\cdot L_{ij}$（9）

Based on equations 4) and (5), the transportation risk on the route$\left(i,j\right)$ can be expressed as:

$TR_{ij}\text{=}\frac{4\theta\cdot Q_{ij}\cdot P_{ij}}{\pi\cdot\mu^{2}\cdot T^{2}+4\mu\cdot T\cdot H}$（10）

综上，运输风险可由下式表示：

Transportation risk is represented by:

$f_{2}=\min\left(\sum_{i,j\in N} TR_{ij}\cdot\mu T\cdot P_{ij}\right)=\min\frac{\sum_{i,j\in N} 4\theta\cdot Q_{ij}\cdot P_{ij}\cdot\mu T}{\pi\cdot\mu^{2}\cdot T^{2}+4\mu\cdot T\cdot H}$（11）

7. The methodology and results sections contain excessive narrative text and insufficient formalism (e.g., pseudocode, mathematical notation). Would the authors consider rewriting key parts using clear pseudocode for the algorithm and structured tables for solution outcomes?

**According to the reviewer’s requirement**, relevant statements have been added to in Section **5.1, 5.2, 6.2, 6.3**.

At this point, the solving steps of the algorithm are as A1.

**Algorithm A1:**

Step1:The initial capacity of the transfer point set is 0, clear the forbidden set of transfer point（）, and put all production points into the set to be allocated.

Step2:Perform cluster analysis on the production points in set, use the minimum envelope method to determine the position of transfer point, and establish the transportation relationship between production point and transfer point.

Step3:If,the transportation relationships between the production points and the transfer point is terminated in descending.

Step4:Put the production points that have been terminated transportation relationships into the set to be allocated and into forbidden set of transfer point.

he genetic algorithm uses natural number encoding to randomly generate an initial population, and the specific calculation stepsof the algorithm are as A2:

**Algorithm A2:**

Step1:Set the population size, maximum number of iteration, crossover probability, and mutation probability.

Step2:Obtain the initial population through clustering, calculate the fitness function based on the objective function, and determine the current algebra.

Step3: Follow the roulette wheel principle to select excellent individuals for inheritance to the next generation.

Step4:Set the crossover probability and mutation probability, obtain a new population and calculate the population fitness.

Step5:Perform a taboo search on the chromosomes, using the generated initial neighborhood solution as the candidate solution set. Update the taboo table for taboo iteration to find the optimal solution, Current Algebra+1.

**Step6:**If the current algebra, and the calculation stops, the optimal solution for the fitness function is the satisfactory solution to the problem**^[22]^**.

By using Matlab to solve the problem, the average distance of each Pareto optimal solution tends to stabilize after about 160 iterations, and the Pareto front is shown in Figure 7 and Table 5.

**Tab.5 Delivery plan results for low medical waste production**

| **Optimal route** | **Path** | **Total**  **Cost** | **Transport**  **risk** | **Vehicle**  **mileage** | **Vehicle**  **Number** |
| --- | --- | --- | --- | --- | --- |
| **First Stage** | Path1_1  Path1_2  Path1_3 | 1063 | 73.12 | 92.62 | 3 |
| **Second Stage** | Path2_1 | 690 | 0 | 18.99 | 1 |
| **Total** |  | 1753 | 73.12 | 111.61 | 4 |

Path1_1 represents : B-17-20-21-22-24-47-48-50-49-27-26-25-23-29-30-33-36-

35-34-32-31-28-19-18-16-B;

Path1_2 represents: B-14-13-10-11-12-7-6-5-4-1-2-3-8-9-15-B;

Path1_3 represents: D-43-44-40-41-42-38-37-46-45-52-51-53-56-55-57-58-54-

39-D;

Path2_1 represents: O-B-D-O.

The medical waste transport route of Pareto solution is shown in Figure 9 and Table 6.

**Tab.6 Delivery plan results for increased medical waste production**

| **Optimal route** | **Path** | **Total**  **Cost** | **Transport**  **risk** | **Vehicle**  **mileage** | **Vehicle**  **Number** |
| --- | --- | --- | --- | --- | --- |
| **First Stage** | A-7-8-5-6-A  A-19-28-30-33-A  B-15-24-22-21-B  B-47-48-49-50-51-B  B-25-27-52-26-B  C-16-20-23-14-C  C-17-18-12-C  D-55-56-53-54-D  D-43-44-40-42-41-D  D-38-37-45-39-D  E-9-3-2-1-E  E-10-13-11-4-E  F-32-36-35-34-31-F  F-46-58-57-29-F | 3249 | 517.26 | 89.78 | 14 |
| **Second Stage** | O-F-D-O  O-C-B-O  O-A-E-O | 1809 | 0 | 30.87 | 3 |
| **Total** |  | 5058 | 517.26 | 120.65 | 17 |

We tried our best to improve the manuscript and made some changes in the manuscript. These changes will not influence the content and framework of the paper. And here we marked in yellow in revised paper. We appreciate for Editors/Reviewers’ warm work earnestly, and hope that the correction will meet with approval.

Once again, thank you very much for your comments and suggestions.
